# Disgust Enhances the Recollection of Negative Emotional Images

**DOI:** 10.1371/journal.pone.0026571

**Published:** 2011-11-16

**Authors:** Camilla J. Croucher, Andrew J. Calder, Cristina Ramponi, Philip J. Barnard, Fionnuala C. Murphy

**Affiliations:** Medical Research Council Cognition and Brain Sciences Unit, Cambridge, United Kingdom; Royal Holloway, University of London, United Kingdom

## Abstract

Memory is typically better for emotional relative to neutral images, an effect generally considered to be mediated by arousal. However, this explanation cannot explain the full pattern of findings in the literature. Two experiments are reported that investigate the differential effects of categorical affective states upon emotional memory and the contributions of stimulus dimensions other than pleasantness and arousal to any memory advantage. In Experiment 1, disgusting images were better remembered than equally unpleasant frightening ones, despite the disgusting images being less arousing. In Experiment 2, regression analyses identified affective *impact* – a factor shown previously to influence the allocation of visual attention and amygdala response to negative emotional images – as the strongest predictor of remembering. These findings raise significant issues that the arousal account of emotional memory cannot readily address. The term impact refers to an undifferentiated emotional response to a stimulus, without requiring detailed consideration of specific dimensions of image content. We argue that ratings of impact relate to how the self is affected. The present data call for further consideration of the theoretical specifications of the mechanisms that lead to enhanced memory for emotional stimuli and their neural substrates.

## Introduction

Memory for emotional events is typically better than memory for comparable non-emotional events. Memories of significant, deeply affecting public events tend to be subjectively vivid and long-lasting, as evidenced by the flashbulb memory phenomenon [1]. Memory is also enhanced for emotional relative to neutral material presented under simplified laboratory conditions, and this effect persists over long intervals [2], [3], [4]. For example, increased retention has been observed for emotional relative to neutral narratives [5], and emotional relative to neutral pictures and words [3], [6].

The dominant theoretical account maintains that the effects of emotion on memory are mediated by arousal, where arousal relates to some physiological state of excitement or activation [7], [8]. Arousal theory as applied to memory is closely coupled to wider dimensional models of the structure of emotion. Dimensional theorists claim that emotional experience can be captured by a small number of orthogonal dimensions [9], [10]. The major affective dimensions are pleasantness (often referred to as valence) and arousal. Whereas the pleasantness dimension ranges from negative to positive, the arousal dimension ranges from calming/soothing to exciting/agitating. Both dimensions have been implicated in emotional memory, though arousal has received particular attention.

Converging evidence from both animal and human studies supports a role for arousal in emotional memory. Animal studies have demonstrated that increased states of arousal improve memory [11], [12], [13], and that the amygdala plays an important role in retention. Most human studies have focused on facilitation of memory for arousing ‘representational’ stimuli, such as pictures of emotional scenes or words [2], [3], [6]. Human studies typically employ emotional and neutral stimuli from databases such as the International Affective Picture Series (IAPS) [14] on the basis of normative subjective ratings of valence and arousal [14], [15], [16], [17], [18].

On the basis of arousal theory, it would be reasonable to predict that increasing levels of arousal would produce corresponding improvements in retention. This hypothesis has received support in some studies that have varied arousal parametrically, while keeping other stimulus attributes constant [17]. However, evidence from multiple sources indicates that the relationship between rated arousal and memory is not always straightforward [19]. For example, Bradley et al. [2] divided emotional images into five categories that ranged from highly unarousing to highly arousing; only highly arousing images produced significantly enhanced memory. Counterintuitive findings have also emerged in younger adults [20], where memory was enhanced for low relative to high arousal negative items. The authors entertained the possibility of a Yerkes-Dodson type of explanation, in which memory is optimum at moderate levels of arousal but reduced at lower or higher arousal levels. However, in a review of the literature, Christianson [21] reported little evidence for the application of the Yerkes-Dodson law to emotional memory.

The emphasis on arousal and valence dimensions within the emotional memory literature has inevitably drawn attention away from the potential influence of other factors, including the basic emotion categories postulated by Darwin [22] and others [23], [24], [25], [26], [27], or indeed, correlates of these categories. A comprehensive review of the literature on emotional memory concluded that findings have been severely limited by the consideration of emotion as ‘merely arousal’, and that a more complete understanding will result only from considering the contributions made by categorical affective states, as well as attributes linked to cognitive appraisal [7].

Though there is variation according to the particular taxonomy, basic emotions have included disgust, fear, anger, happiness, sadness, and surprise. Some of these have very similar profiles in the dimensional model, but nonetheless distinct qualities, and in some cases, distinct neural bases [28]. Fear and disgust are of particular significance for evaluating the effects of arousal as neurophysiological evidence indicates that the two are associated with partially distinct neural substrates [28], [29]. Furthermore, psychophysiological evidence and behavioural ratings indicate that fear is a high arousal unpleasant emotion, whereas disgust is a moderate arousal unpleasant emotion [30], or certainly no more arousing than fear.

If arousal is causal, then a straightforward prediction would be that memory for disgusting stimuli should be worse, or certainly no better than memory for frightening stimuli of equal unpleasantness. However, there are reasons to suppose that this might not be the case. Charash & McKay [31] failed to find a recall advantage for fearful words, reporting a recall advantage for disgusting over frightening words instead. The conclusions drawn from that study were not entirely persuasive as the disgust, threat, and neutral word lists were matched for word frequencies but not for affective variables such as pleasantness and arousal. Nevertheless, additional research has shown that another factor known to influence recollection – attention to the to-be-remembered material at encoding [32], [33], [34] – is associated to a greater extent with disgust than with fear-related stimuli. Relative to frightening stimuli, disgusting stimuli more readily engage interest and attention during the first 500 ms of viewing, demonstrated using eye-tracking methodology [35].

Another key issue in the emotional memory literature surrounds the extent to which emotion has dissociable influences on distinct memory processes. In an elegant study, Ochsner [17] employed the Remember/Know paradigm to study recognition memory for pictorial emotional stimuli. The term remembering refers to a positive recognition response that is accompanied by recollection of the encoding context, such as thoughts, feelings, and sensory details that were experienced when the stimulus was first presented. The term knowing, on the other hand, refers to a positive recognition response that is associated with the knowledge that the stimulus is familiar and has been seen before but without any recollection of the episodic context. Whereas previous research had shown that highly arousing or highly negative or positive materials are better retained than neutral ones [2], Ochsner [17] demonstrated that these effects are most apparent for measures of recollection rather than familiarity, and that both pleasantness and arousal have significant, independent effects on recollection.

Given that investigations of emotional memory will benefit from consideration of categorical affective states, it seemed important to revisit this topic using well-controlled stimuli. The current research compared memory for images of equally negative disgusting and frightening scenes; this addressed the role of arousal directly, as there is reason to expect that lower-arousal disgusting images may be better recollected than higher-arousal frightening ones. In addition to matching the two image sets for pleasantness, it was also considered important to match them for distinctiveness, as distinctive items are often well-remembered [36], [37], [38] and for visual complexity, an attribute used to match stimulus categories in previous studies of emotional memory [17], [18]. Following the lead of Ochsner [17] and Kensinger & Corkin [6], the Remember/Know procedure was employed to assess recognition memory and to derive estimates of recollection- and familiarity-based memory. On the basis of previous findings [6], [17], we predicted that any effects of emotional content would be particularly evident in estimates of recollection for the episodic context.

## Results and Discussion

### Experiment 1: familiarity and recollection of disgusting versus frightening images

Experiment 1 compared recollection and familiarity for images of disgusting, frightening, and mildly positive scenes. The latter were used as a control, in preference to neutral scenes, so that the three image categories contained some emotional meaning. The use of a positive image category as a baseline condition also enabled us to match baseline and negative image categories on a key content attribute, the presence of people, as most neutral images in the IAPS feature only objects or buildings. As noted above, the Remember/Know procedure was employed to assess recognition memory. The dependent measures were estimates of recollective memory, calculated from remember hits and false alarms, and familiarity-based memory, calculated from know hits and false alarms [39]. Our prediction was that recollection estimates for disgusting images would be significantly greater than for frightening ones.

A repeated-measures ANOVA on participants' ratings of image redness in the encoding session showed no significant effect of image category, *F* (1.42, 42.72) = 2.33, *p*>0.1 (Greenhouse-Geisser corrected). Hence, any memory differences cannot be attributed to differences between the stimuli on the feature judged at encoding.

Mean recollection and familiarity estimates for the disgusting, frightening, and positive images are shown in Table 1. Proportions of hits and false alarms for the remember and know responses are also shown. All post hoc comparisons were Bonferroni corrected for multiple comparisons, with corrected *p* values reported throughout unless stated otherwise.

**Table 1:** Mean memory scores from Experiment 1, by image category (disgust, fear or positive).

	Disgust	Fear	Positive
Recollection estimate	0.41 (0.20)	0.31 (0.20)	0.25 (0.18)
Familiarity estimate (*fd*′)	1.37 (0.76)	0.85 (0.73)	1.13 (0.74)
Remember hits	0.43 (0.20)	0.35 (0.20)	0.28 (0.18)
Remember false alarms	0.04 (0.06)	0.05 (0.08)	0.04 (0.08)
Know hits	0.30 (0.19)	0.27 (0.14)	0.35 (0.14)
Know false alarms	0.08 (0.10)	0.13 (0.09)	0.10 (0.11)

Standard deviations are shown in brackets. There was a significant recollection advantage for disgusting images compared to the other categories (*p*'s<0.005), and a parallel effect for familiarity estimates.

A repeated measures ANOVA examining the effects of image category (disgusting, frightening, and positive) on recollection estimates showed a significant main effect, *F* (2, 62) = 15.43, *p*<0.001, *MSE* = 0.01, *η_p_* ^2^ = 0.33. Recollection estimates for disgusting images were significantly greater than both frightening images, *t* (31) = 3.74, *p*<0.005, *d = *0.50, and positive images, *t* (31) = 4.86, *p*<0.005, *d = *0.84. The difference in recollection estimates between frightening and positive image categories was not statistically significant, *p* = 0.09.

Familiarity estimates data were submitted to an identical ANOVA. The effect of image category was significant, *F* (2, 62) = 6.85 *MSE* = 0.31, *p*<0.001, *η_p_* ^2^ = 0.19, reflecting higher *fd′* scores for disgusting relative to frightening images, *t* (31) = 3.79, *p*<0.005, *d = *0.70. No other comparisons were significant (*p*>0.10).

The findings are consistent with recent results indicating that disgusting images preferentially attract attention when presented simultaneously with frightening images [35]. They also indicate that the previous advantage for disgust-related words [31] in a recall memory experiment extends and generalises to recollection and familiarity estimates for disgusting images that are matched carefully with frightening images for pleasantness, approach-avoidance, distinctiveness, visual complexity, anger and sadness.

Perhaps most importantly, the current findings of increased memory for disgusting relative to frightening images raise questions about accounts of emotional memory effects that rely on arousal as the primary explanatory factor. The disgusting images were rated as significantly *less arousing* than the frightening images, and although this difference was small, the arousal account would certainly not predict improved recognition memory for disgust relative to fear images. Similarly, increased memory was observed for the disgusting relative to positive control images, even though these two stimulus categories were matched for rated arousal. Thus, under circumstances where tightly controlled materials were employed, the predictions of the theory linking arousal and memory were not supported.

The memory advantage for disgusting relative to frightening images was found for both recollection and familiarity. By contrast, previous work has shown the influence of pleasantness and arousal on emotional memory to be primarily on recollection estimates [17]. In addition, whereas Ochsner [17] found that negative images were recollected more readily than positive images, Experiment 1 found that the negative image advantage was greater for disgusting than for frightening images, despite both image sets being matched for pleasantness, and negative valence in particular. Thus, the memory advantage for disgusting images cannot be attributed to variation in this dimension either and must instead derive from attributes other than valence and arousal.

Our data so far suggest that not all arousing images have a strong enough effect upon participants to result in significantly increased recollection relative to carefully-controlled stimulus sets; therefore factors other than arousal and valence need to be identified and integrated into causal accounts of emotional memory. This idea is consistent with the results of a recent review of the emotional memory literature [7] in which the contributions of categorical affective states (e.g. basic emotions) and attributes based in cognitive appraisal theory were considered essential to an understanding of emotional memory. It has been similarly suggested that emotional stimuli engage semantic information and appraisal processes [17] as well as incorporate non-emotional attributes [40] that could affect memory in ways that remain to be investigated. Experiment 2 explored dimensions other than valence and arousal that might account for the enhanced retention of disgusting images.

### Experiment 2: exploring dimensions that may account for enhanced memory of emotional images

It is possible that our disgusting stimuli were more memorable simply because they related to disgust. Another possibility is that our disgusting images might have been more memorable because they weigh particularly heavily on psychological attributes or processes that facilitate retention – these attributes or processes would not be considered to be specific to the emotion disgust, but to also contribute to the memory advantage observed for emotional stimuli more generally.

Memory researchers and appraisal theorists have suggested a range of plausible influences on memorability. Together, they have identified a range of attributes that are important in the generation of varied emotional experiences and reactions [41]. These include, but are not limited to, salience, thematic relevance to the self, incongruity, meaningfulness, and importance. Given the presence of strong correlations between at least some of these factors, notably importance (i.e. consequentiality), meaningfulness and memorability [42], what the constructs share is plausibly more significant than the specifics of the individual dimensions identified.

We have previously argued that attributes such as those identified as important in the generation of emotional experience, may contribute, individually or collectively, to a factor that has been the focus of recent empirical investigations – the immediate *impact* an item has on an individual [43], [44]. The term impact derives from photojournalism where it is used to describe powerful and striking images [45]. In recent behavioural and neuroimaging studies, impact has been shown to influence the allocation of visual attention [44] and also the amygdala response [43] to negative emotional images, to high versus low impact image sets that have been carefully matched on a number of stimulus attributes, including arousal and valence. These studies argued that ratings of impact reflect an individual's undifferentiated reaction to the image and thus index the immediate significance or relevance of a stimulus for the self. Given that increasing or dividing attention to encoded information is known to lead to enhanced and reduced remembering, respectively [32], [34], [46] and that the amygdala is a brain structure known to be important for emotional memory [47], [48], [49], [50], [51], [52], it follows that ratings of impact may be a strong predictor of emotional memory in the present study.

A preliminary visual inspection of the most and least frequently remembered images across our image categories suggested that the former did indeed have immediate and strong effects upon the viewer, providing some initial support for this idea. This effect was not limited to the disgusting images, however, as several frightening images that were frequently remembered also appeared to share this eye-catching quality. As noted above, the impact of these images upon the viewer could relate to a number of different factors such as the extent to which the images were incongruent with participants' previous experience, such that their meaning or significance was in one way or another difficult to grasp. One example is a picture of a man kissing the side of a woman's head. The woman is bruised and bloodied and appears to be unconscious or dead. It is unlikely that participants would have seen an image like this previously. Thus, the image as a whole is incongruous with previous experience, makes a strong impression, and is consequently, well-remembered.

The purpose of Experiment 2 was to determine which attributes are the strongest predictors of the emotional memory effects observed in Experiment 1 by having participants rate the stimuli on a number of dimensions. To explore the contribution of impact, identified in previous research as a key determinant of heightened attention and amygdala response to negative emotional images, we employed an impact rating scale; this scale captured participants' undifferentiated response to images as indexed by the immediate impact these images had upon the participant. In the Introduction we noted that ratings of arousal may relate to some physiological state of excitement or activation. As it is possible that ratings of impact may likewise have a physiological basis, we also included ratings of participants' negative and positive body state reactions to the images. Both Tulving [53] and Gardiner [54], [55] have argued that remember responses are often based on remembered thoughts and feelings. To assess this sort of elaborative processing, we included a fourth rating scale that indexed the number of thoughts and ideas evoked by the content of each image. These four new ratings (impact, negative and positive body state reactions, and ideation) supplemented our earlier ones (arousal, pleasantness, approach-avoidance, distinctiveness, and visual complexity) to enable us to determine which factors best predict picture recognition. The prediction was that images that were frequently remembered in Experiment 1 would be rated high in impact by this separate group of participants and that ratings of body state and ideation might relate to judgements of impact.

A summary of mean ratings, and Mann-Whitney comparisons between the three image categories employed, is shown in Table 2. It was not considered appropriate to calculate recollection estimates in an items analysis as different participants contributed remember hits and remember false alarms. Instead, stepwise multiple regression analyses were conducted to isolate the variables that predicted *remember hit rates*. Indeed, in Experiment 1, the correlation between recollection estimates and remember hit rates was extremely high, *r* (32) = 0.97, *p*<0.001. The predictors entered into the multiple regressions included the four personal reaction ratings (impact, negative and positive body state reactions, and ideation), and also the five previously collected ratings (arousal, pleasantness, approach-avoidance, distinctiveness, and visual complexity).

**Table 2:** Mann Whitney U tests on Personal Reaction ratings for the Experiment 1 image categories.

	Disgust	Fear	Positive	Disgust vs. Fear	Disgust vs. Pos	Fear vs. Pos
	Mean (*SD*)			Mann-Whitney U (z)		
Impact	5.68 (1.81)	4.49 (1.34)	2.90 (1.26)	395.00 (−2.87)*	141.00 (−5.73)***	259.00 (−4.41)***
Neg. body state	6.36 (1.87)	6.00 (1.25)	1.03 (0.12)	534.50 (−1.29) NS	0.00 (−7.72)***	0.00 (−7.73)***
Pos. body state	1.01 (0.08)	1.11 (0.30)	6.39 (1.27)	574.50 (−1.73) NS	0.00 (−7.77)***	0.00 (−7.62)***
Ideation	3.49 (1.28)	3.72 (1.08)	3.50 (0.82)	561.50 (−0.99) NS	616.00 (−0.37) NS	580.00 (−0.78) NS

Bonferroni corrected, asymptotic two-tailed significance levels are denoted as follows:

***p<0.005;

**p<0.01,

*p<0.05.

Across the disgusting, frightening and positive images, the best regression model had an Adj*R* ^2^ of 0.30, *F* (2, 105) = 22.00, *p*<0.001. There were two significant predictors of remember hit rates: impact (beta = 0.33, *p*<0.001) and distinctiveness (beta = 0.26, *p*<0.05). However, when only the negative (disgusting and frightening) images were included in a similar analysis, impact was the only predictor: this model had an Adj*R* ^2^ of 0.32, *F* (1, 70) = 33.53, *p*<0.001. Impact was a highly significant predictor, with a beta of 0.57, *p*<0.001. The latter analysis therefore suggests that the contribution of distinctiveness to remembering was attributable to the inclusion of the less distinctive (and less well remembered) positive images.

Crucially, arousal ratings *did not* emerge as a predictor of remembering. This suggests that the memory effects attributed to differences in arousal ratings in previous investigations [2], [17] may in fact have resulted from other factors.

Further stepwise multiple regressions were conducted to discover what image characteristics were the strongest predictors of impact ratings. A first analysis examined the disgusting, frightening and positive images. The independent variables specified above were entered, with the exception of impact, which constituted the dependent variable. The resultant model was highly significant, Adj*R* ^2^ = 0.64, *F* (4, 103) = 48.40, *p*<0.001, and revealed that four aspects of the Experiment 1 images were important contributors to their impact level. Negative body state reaction was the strongest predictor of impact (beta = 1.07, *t* = 7.20, *p*<0.001), followed by positive body state reaction (beta = 0.57, *t* = 4.41, *p*<0.001) and distinctiveness (beta = 0.29, *t* = 0.29, *t* = 3.59, *p*<0.005). The fourth predictor in this model was ideation, and this rating was the only *negative* predictor of impact (beta = −0.16, *t* = −2.28, *p*<0.05). A similar multiple regression for disgust and fear images alone showed an Adj*R* ^2^ of 0.57, *F* (3, 68) = 32.83, *p*<0.001. The predictors were negative body state reaction ratings (beta = 0.57), distinctiveness (beta = 0.42), and ideation (beta = −0.21), *p*'s<0.05. Thus, the contribution of positive body state reaction to the previous model reflected the inclusion of positive images.

Arousal was not identified as a significant predictor in any of the regression models. Thus, arousal, conceived by many dimensional theorists as an “excitement” dimension and associated with physiological arousal, does not fully capture either the affective properties of emotional memory or the conceptual basis of impact. Importantly, the multiple regression analysis does not discount some association between arousal and impact; indeed, impact was found to significantly correlate with arousal, *r* (108) = 0.45, *p*<0.001. Rather, it shows that image properties other than arousal are more important in determining impact.

### General Discussion

The goal of this research was to examine the influence of disgusting versus frightening negative emotional images on recognition memory and to explore factors that contribute to heightened recollection. The results of Experiment 1 indicated that disgusting scenes were retained to a greater extent than images of equally unpleasant frightening scenes, with larger recollection estimates calculated for disgusting scenes. Importantly, this pattern emerged even though the disgusting images were rated as less arousing than the frightening ones but were otherwise matched for unpleasantness, distinctiveness, visual complexity, and other stimulus attributes. In Experiment 2, multiple regression analyses showed that improved recollection was best accounted for by differences in the immediate *impact* of the images, and not differences in arousal or pleasantness.

These data indicate that the memory advantage for disgusting images may derive from attributes other than valence and arousal, dimensions that feature prominently in the emotion memory literature [17], [40], [56]. Contrary to the widely held view that arousal in particular is a major determinant of emotional memory effects, the present data suggest that a construct that we refer to as ‘impact’ may offer a more adequate explanation. A role for impact aligns with our previous work showing that impact influences the allocation of visual attention to negative emotional images [44], as increased attention to encoded information is known to enhance recollection [32] whereas divided attention is known to reduce recollection [34], [46]. It is furthermore consistent with research showing a heightened amygdala response to high versus low impact negative images [43], as both lesion and functional neuroimaging studies [47], [48], [49], [50], [51], [52] have demonstrated that the amygdala plays a central role in emotional memory. The current findings also correspond with research showing that adolescents' and adults' appraised *impact* of the September 11^th^ US attacks predicted recalled intensity of sadness, anger, and anxiety, changes in memory for these emotions over time, and symptoms of post-traumatic stress [57].

Participants' ratings of impact indicated the extent to which they were affected by the images, that is, how much they felt the image content created an instant impact on them personally. Our procedure for rating impact used short presentation durations, and the instructions emphasized immediate judgments based on generic senses or feelings [58] without deconstruction of their elements. In previous work [43], [44], we have argued that impact ratings index participants' undifferentiated emotional response to a stimulus, without requiring more detailed consideration of specific dimensions of image content. To illustrate, an individual might have an undifferentiated ‘Yuk’ or ‘What the …?’ reaction to a specific image, without explicit consideration of how disgusting or arousing that image might be.

This is relevant to a distinction that has been drawn between emotional images inducing a genuine emotional reaction (e.g., making you feel ‘sick to your stomach’), and those being coldly appraised as having ‘affective quality’ (e.g. a cold evaluation that an image depicts a nauseating scene) [58]. It is possible that arousal and pleasantness ratings failed to predict memory well because these ratings can be more readily made on the basis of intellectually-detached judgments of affective qualities. In contrast, rated impact may have been a stronger predictor because it relies on some degree of personally felt core affect, however fleetingly invoked in a laboratory setting. Rusell [10] has defined *core affect* as a neurophysiological state that is ‘consciously accessible as a simple, nonreflective feeling that is an integral blend of hedonic and arousal values’. Insofar as ratings of impact were designed to quantify a genuine emotional response, it is noteworthy that negative body state reactions were prominent in the regression analysis of impact ratings. Arousal, on the other hand, was not. This indicates that the impact ratings are capturing at least some qualitative attributes of visceral, and potentially other bodily reactions that are missed by assessments of arousal in the current study. The observed involvement of negative body state reactions is not unexpected as it is consistent with theoretical work proposing that disgust is an emotion that evolved from a more primitive system involved in distaste [59]. Indeed, it has been suggested that disgust is often experienced as a visceral sensation, and that this is likely to be due to the processes of nausea, throat clenching, and food expulsion that are often triggered by this emotion [60]. Schnall and colleagues [60] further suggest that whereas emotions typically involve a physical and embodied component, this feature may be particularly pronounced for the emotion disgust.

Though impact ratings were designed to assess the immediate emotional effects of the image upon the viewer rather than an intrinsic property of the image, these ratings are likely to incorporate and be influenced by attributes identified by appraisal theorists as important in the generation of emotional reactions and responses [41]. These include attributes such as the immediate significance or relevance of a stimulus for the self, and distinctiveness or incongruity. Relevance to the self relates to constructs such as core affect, discussed above, and the “working model of the self” [61] or “schematic model of the self” [62] discussed in relation to autobiographical recollection and Remember/Know performance in sad mood states, respectively. Personal relevance is known to contribute to flashbulb memory formation in the case of emotionally-charged public events [1], and influences whether an event is remembered rather than known [63]. Interestingly, though Ochsner [17] found a recollection advantage for negative relative to positive images matched for arousal, he noted that this advantage might have been lost had the two image sets been equated for ‘personal relevance.’ Furthermore, Adolphs [64] has argued that ‘constructs such as impact and relevance’ should form the basis for investigating individual differences in amygdala function. This interpretation accords with Ewbank's conclusion that amygdala function is determined not simply by arousal (or valence) alone but by an event's significance or relevance to the individual [43].

Further relevant to the current research is the contribution of distinctiveness to rated impact and estimates of recollection. Though the memory advantage for disgusting relative to fearful stimuli emerged despite these stimulus sets being matched for distinctiveness, after body state reactions, distinctiveness was a second predictor of impact ratings. As operationalised in our study, items can be distinctive on the basis of semantic through to perceptual levels of analysis. Thus, images having impact seem to involve an intersection between felt affect and more traditional cognitive attributes associated with what makes events distinct, such as rareness or incongruity with prior experience. In the literature examining memory for non-emotional and emotional items, distinctive items tend to be remembered or recalled more often than less distinctive items [36], [37], [38]. It is important to emphasise that distinctiveness is not equivalent to visual complexity, which has been used to match stimulus categories in previous studies of emotional memory [17], [18] but did not emerge as a significant predictor of impact or recollection here.

The present findings demonstrate that disgusting images may be better remembered than equally pleasant frightening ones that are matched for a range of emotional and non-emotional stimulus attributes, despite the disgusting images being less arousing. Neither arousal- nor valence-based accounts of emotional memory can readily account for this finding, with the construct of impact instead emerging as a strong predictor of the current emotional memory effects. Previous work has shown that impact influences the allocation of visual attention and the amygdala response to negative emotional images, yet important issues remain to be addressed. One obvious application of the present findings would be to assess memory for well-controlled stimuli that vary in rated impact, while another would be to assess whether impact's explanatory power extends to memory for highly positive images and stimuli in other modalities. The present contribution of rated body state, distinctiveness and (lack of) ideation to impact further suggests promise in clarification of the factor structure that underlies diverse ratings of image content. In the case of the bodily state reactions to high impact images, examination of psychophysiological indices should also be prioritised.

It should be noted that a potential limitation of this research is that regression analyses were conducted using memory data and stimulus ratings collected from separate samples of participants. Memory was not tested in Experiment 2 because multiple viewings and ratings might have biased recollection even two weeks later. Though the current data leave open the issue of whether participants' own ratings would predict memory in a way similar to that described here, previous research has reported that for rated impact, consistency of ratings across participants is highly significant [43], [44]. It would therefore not be unreasonable to expect a similar pattern of findings if both datasets had been drawn from the same sample of participants.

Overall, our findings are consistent with the idea that the extant research on emotional memory has been constrained by its treatment of emotion as merely arousal. They demonstrate that a more complete understanding will only result from systematic consideration of the contributions made by categorical affective states (e.g. basic emotions) and other stimulus and event attributes linked to cognitive appraisal [7]. More specifically, the present research suggests a role for the appraised impact of emotional images. While the concept of impact has not been evaluated in experimental memory research, it has played a key role in artistic discourse. A carefully-crafted media photograph should be indelible over long periods, and in photojournalism, striking and eye-catching visual images are routinely referred to as images with impact [45].

## Methods

### Experiment 1

This research was approved by the University of Cambridge Psychology Research Ethics Committee and was conducted according to principles expressed in the Declaration of Helsinki.

#### Participants

Participants gave informed written consent before participating. Thirty-two community volunteers (twenty-five females; mean age = 35.2, *SD* = 7.9) participated in exchange for a small honorarium. All had normal or corrected-to-normal vision.

#### Stimuli

The stimulus set comprised 36 disgusting, 36 frightening, and 36 positive 72 dpi colour photographic images. Images were selected on the basis of ratings from a larger set of 208 images, the majority of which were taken from the International Affective Picture System (IAPS) [14]. All images had been rated previously for five basic emotions (disgust, fear, happiness, anger and sadness) and five additional variables (pleasantness, arousal, tendency to approach or avoid, distinctiveness, and visual complexity). The instructions had emphasized that participants should rate the images on the basis of their own personal reactions rather than how people in general should feel. The ratings on these other dimensions made use of well-established scales. The instructions for rating pleasantness and arousal were described according to the descriptions in the IAPS manual [14]; approach-avoidance was rated with endpoints *very inclined to approach the scene* to *very inclined to avoid the scene*; distinctiveness was rated on the basis of how rarely similar scenes are encountered, relative to other scenes or images, in everyday life; and visual complexity instructions were adapted from Ochsner [17]. More specifically, an image could be considered complex either because it had many simple objects that each had little detail, or a few objects that each had a lot of detail. Lower scores on the 9-point likert scales indicated unpleasant, low arousal, high approach, indistinctive, and low visual complexity image qualities.

The mean ratings for the three image categories employed in Experiment 1 are given in Table 3. Mann-Whitney comparisons showed that the frightening images were rated as more frightening than the other two categories, the disgusting images as more disgusting, and the positive images as more pleasant. Frightening images were also rated as the most arousing, whereas disgusting and positive images did not differ on arousal ratings. Disgusting and frightening images were matched on the following other dimensions: pleasantness, approach-avoidance, distinctiveness, visual complexity, anger and sadness. The stimuli were further divided into two closely matched study sets (set one and set two) on the basis of their content and ratings, each consisting of 18 images belonging to each emotion category. Participants viewed one study set in the encoding session, while the other set served as foils for the recognition memory test. Sixteen mildly unpleasant filler images were also included to minimize primacy and recency effects.

**Table 3:** Mann-Whitney U tests, comparing ratings on the Experiment 1 image categories.

	Disgust	Fear	Positive	Disgust vs. Fear	Disgust vs. Pos	Fear vs. Pos
	Mean (*SD*)			Mann-Whitney U (z)		
Disgust	4.65 (1.48)	1.58 (1.33)	1.00 (0.00)	44.00 (−6.96)***	0.00 (−7.83)***	414.00 (−3.94)***
Fear	1.24 (0.63)	5.14 (1.18)	1.00 (0.00)	1.00 (−7.57)***	522.00 (−2.76)*	0.00 (−7.84)***
Happiness	1.00 (0.00)	1.00 (0.00)	6.12 (0.93)	648.00 (0.00) NS	0.00 (−7.88)***	0.00 (−7.88)***
Anger	2.02 (1.66)	1.69 (1.10)	1.00 (0.00)	631.00 (−0.22) NS	432.00 (−3.75)***	396.00 (−4.11)***
Sadness	2.46 (2.17)	2.23 (1.75)	1.00 (0.00)	626.00 (−0.29) NS	396.00 (−4.11)***	414.00 (−3.93)***
Arousal	4.12 (1.34)	4.86 (1.09)	4.08 (0.92)	382.50 (−3.02)**	589.00 (−0.67) NS	346.50 (−3.44)***
Pleasantness	1.97 (0.68)	2.01 (0.59)	7.51 (0.72)	591.50 (−0.70) NS	0.00 (−7.40)***	0.00 (−7.47)***
Approach/avoidance	7.08 (1.07)	7.54 (0.78)	2.52 (0.98)	456.00 (−2.23) NS	3.00 (−7.32)***	0.00 (−7.36)***
Distinctiveness	5.26 (1.91)	5.47 (1.46)	3.38 (1.31)	585.00 (−0.72) NS	285.00 (−4.12)***	192.50 (−5.18)***
Vis. Complexity	4.15 (1.28)	3.85 (1.36)	4.08 (1.02)	581.00 (−0.76) NS	637.00 (−0.13) NS	583.00 (−0.74) NS

Bonferroni corrected, asymptotic two-tailed significance levels are denoted as follows:

***p<0.005;

**p<0.01,

*p<0.05.

#### Design

The independent variable was image category (disgusting, frightening, and positive images; repeated measure). The experiment consisted of an encoding session and a test session separated by an interval of approximately two weeks (mean interval = 13.75 days, *SD = *1.02). Half of the participants were shown study set one as targets while study set two served as foils in the test session; for the other half of participants the target and foil sets were reversed. The dependent variables in the test session were the proportions of remember, know and new responses made to targets and foils from each image category. These responses were converted into recollection and familiarity (*fd′*) estimates, using the equations shown in Appendix S1.

#### Procedure

The images were presented on a VDU using PsyScope [65]. They were presented against a black background, and subtended a vertical visual angle of approximately 15° and a horizontal visual angle of approximately 13°.

In the encoding session, participants were presented with 18 disgusting, 18 frightening, and 18 positive images from stimulus set one or set two. Images were shown in one of four pseudorandom orders for each stimulus set across three counterbalanced blocks, such that no more than three images from the same emotion category were shown consecutively. To minimize primacy and recency effects, eight filler images were presented at the beginning and end of the encoding session. On each trial, a fixation cross was presented for 500 ms, followed after a 500 ms inter-stimulus interval (ISI) by an image displayed for 5000 ms. Participants were instructed to rate each image for how much red it contained, and to do this from the perspective of a picture editor of a journal, because red did not reproduce well at the printers. This served to focus attention on each image, without requiring any in-depth processing of their emotional or semantic content. Participants were not aware that their memory would be tested when they returned approximately two weeks later for the test session.

In the test session, participants were initially trained in the Remember/Know distinction, with a procedure adapted from Gardiner, Ramponi and Richardson-Klavehn [66]. They were asked to indicate using a button box whether the image had been presented in the encoding session and they could recollect details about the context in which it had been presented (“remember”), or whether the image seemed familiar but they could not recollect anything about the context in which the image had been presented at encoding (“know”), or whether the image had not been presented at encoding (“new”). Guessing was strongly discouraged. Participants viewed images from the study set intermixed with images from the image set that was not presented at encoding (foils). Each image remained on the screen until the participant made their response. Images were presented in four pseudorandom blocks whose order was counterbalanced across participants.

### Experiment 2

#### Participants, design, and procedure

A new group of 12 participants (8 women; mean age 31.50 years, *SD = *6.37) rated the full set of 208 images from which the Experiment 1 images had been selected, for four variables – impact, negative body state reaction, positive body state reaction, and ideation. The images were presented on a VDU, in the same way as the encoding session of Experiment 1. The instructions emphasized that participants should rate the images on the basis of their own personal reactions rather than how they imagined people in general should feel. All participants first rated the images for their immediate impact following a short (500 ms) presentation on a scale that ranged from 1 (no impact) to 9 (intensive impact). Following Murphy et al. [44], participants were instructed to consider each picture as a whole and judge whether they felt the content of the image created an instant sense of impact on them personally. They were asked not to think in detail about the picture or its contents in terms of particular properties like the positive or negative feelings it might invoke (e.g. joy, anger, etc.), how distinctive the image was or how many thoughts and ideas it led to.

The instructions for rating impact were as follows: “In this experiment you will view a series of pictures with varying content. Each will be presented only for a very short amount of time – this is because we want you to rate each one for its immediate impact. By this we mean that before you get to think about what is in the picture you may be instantly affected by it – without necessarily knowing why. We would like you to consider each picture as a whole. Just judge whether you feel the content of the image created an instant sense of impact on you personally. Try not to think in detail about the picture or its contents in terms of particular properties like the particular positive or negative feelings it might invoke in you (e.g. fear, anger, joy, etc.), how distinctive the image is or how many thoughts and ideas it leads to. We just want an estimate of its overall immediate impact, irrespective of what it is that might underlie its impact on you personally (i.e. whether it's positive, negative or neither). Remember, it is your own personal reaction we are interested in, not how you think people in general should feel. Just glance at the picture and make an ‘instant’ judgment.” No participant reported difficulty with understanding the impact rating instructions, and importantly, a high level of agreement in impact ratings across participants has been reported previously [43].

Following the impact rating task, the images were viewed again, this time for 5000 ms each, in a different order, and each image was then rated for (1) negative body state reaction, (2) positive body state reaction, and (3) ideation (the number of elicited thoughts and ideas). The order of the scales was counterbalanced. The former two contrast with pleasantness ratings by emphasising genuine visceral and emotional feelings, as opposed to cold rational appraisals. For negative body state reaction, participants were asked to indicate the extent to which each scene caused a negative body state reaction such as an unpleasant feeling in the pit of their stomach, shivers up their spine, or hairs on the back of their neck standing on end. For positive body state reaction, participants were asked to indicate the extent to which each scene caused a positive body state reaction such as feeling warm inside, wanting to laugh out loud, or feeling so happy that they might cry. Ratings of negative and positive body state reactions were done on 9-point scales with endpoints 1 *no negative/positive body state reaction* to 9 *very high negative/positive body state reaction*. For ideation, participants were asked to rate how many thoughts and ideas came to mind for each image on a scale with endpoints (1 *none at all* and 9 *a large number of ideas*).

## Supporting Information

Appendix S1 Equations used to calculate the two main dependent variables, Recollection scores and Familiarity scores.
(DOC)
