# Supplementary material for: Disgust Enhances the Recollection of Negative Emotional Images
Source: PLoS One. 2011 Nov 16;6(11):e26571. doi: 10.1371/journal.pone.0026571 (PMC3217922; doi:10.1371/journal.pone.0026571)
Supplement: Appendix S1 — Equations used to calculate the two main dependent variables, Recollection scores and Familiarity scores. (DOC) [file pone.0026571.s001.doc]

Appendix S1. Equations used to calculate the two main dependent variables, Recollection scores and Familiarity scores. Recollection estimates were calculated according to the equation published by Yonelinas et al. [39]. In the equation, R(old) denotes numbers of Remember hits, and R(new) denotes numbers of Remember false alarms. Each is converted into proportions by dividing by N, the number of opportunities to give an R response.

ROLD_ - RNEW_

N N

Recollection = _________________________________

1 - RNEW_

N

The familiarity (*fd’*) equation was adapted from Yonelinas et al. [39], on the advice of a statistician. Foil items may be falsely recognised on the basis of feelings of familiarity, and so the familiarity of target items, F(old), and the familiarity of foil items, F(new), are calculated separately. The dependent variable *fd’* is equal to the distance between the F(old), or signal, and F(new), or noise, distributions. In these equations, numbers of Know hits are denoted as K(old), and numbers of Know false alarms as K(new). The calculations were smoothed, by adding 0.5 to the numerator and 1 to the denominator, to avoid the missing values that would result if K(old) or K(new) were equal to zero.

| F_OLD_ = | ( K_OLD_ + 0.5 ) |
| --- | --- |
|  |  |
|  | (K_OLD_ + MISSES + 1) |
|  |  |

| F_NEW_ = | ( K_NEW_ + 0.5 ) |
| --- | --- |
|  |  |
|  | ( K_NEW_ + HITS + 1 ) |
|  |  |
